# Potent P2Y_12_ Receptor Inhibitors Reduce Risk for Hospitalization from Sepsis in Patients with CKD

**DOI:** 10.34067/KID.0000000918

**Published:** 2025-07-21

**Authors:** Nishank Jain, Lucas R. Johnson, Dharmik Jadvani, Prabin Shrestha, Csaba P. Kovesdy

**Affiliations:** 1 Department of Internal Medicine, University of Arkansas for Medical Sciences, Little Rock, Arkansas; 2 Division of Nephrology, University of Tennessee Health Science Center, Memphis, Tennessee; 3 Nephrology Section, Memphis Veterans Affairs Medical Center, Memphis, Tennessee

**Keywords:** chronic inflammation, CKD, clinical epidemiology, platelets

In the last two decades, the all-age death rate for CKD nearly doubled worldwide.^1^ This death rate is much higher than the all-age death rates for diabetes, chronic obstructive pulmonary disease, and depressive disorders.^1^ Thus, novel treatments are needed to prevent death of patients with CKD.

One way to prevent death of patients with CKD is to prevent lethal sepsis, a common cause of death for people with CKD.^2^ Sepsis develops from one or more adverse host responses to infection and develops more commonly in those with chronic diseases such as CKD.^3^ Sepsis activates circulating platelets, and in turn, these platelets modulate immune cell phenotype and orchestrate the inflammatory dysregulation associated with sepsis.^4^ Preventing inflammation that arises from activated circulating platelets may reduce the risk of sepsis or its severity for patients with CKD.

Platelet-driven inflammation may be caused by activation of the purinergic receptor P2Y_12_, a receptor found primarily on platelets. Activation of P2Y_12_ leads to platelet degranulation which modulates leukocytes for production of proinflammatory cytokines, *e.g*., IL1*β* and TNF*α*, implicated in sepsis^4^; by contrast, inhibition of P2Y_12_ decreases cytokines, which in turn could decrease inflammation in patients with CKD.^4,5^ Therefore, inhibition of P2Y_12_ may prevent or reduce the severity of sepsis by decreasing the severity of the inflammatory response. Initial findings showed that when compared with a less potent P2Y_12_ inhibitor (P2Y_12_-I), potent P2Y_12_-Is were associated with lower risk for lethal sepsis in dialysis patients with ESKD.^6^ Whether this finding translates to patients with CKD who are not receiving dialysis is unclear.

To address this question, we performed a retrospective study to determine whether sepsis in nondialysis patients with CKD was better controlled by the potent P2Y_12_-Is prasugrel or ticagrelor than by the less potent P2Y_12_-I clopidogrel. We used the Therapeutic Interventions in CKD cohort, a retrospective cohort of 3,562,882 US Veterans who had normal kidney function when enrolled in the cohort (2004–2006), and followed until September 30, 2019. From this cohort, we selected Veterans who developed incident CKD. We defined incident CKD as an eGFR of <60 ml/min per 1.73 m^2^. This eGFR had to be recorded at least twice with more than 90 days between eGFR tests, and the eGFR had to be at least 25% lower than the eGFR determined for the person during cohort enrollment. We then determined which of these patients received new prescriptions for potent P2Y_12_-Is or less potent P2Y_12_-I clopidogrel after the CKD diagnosis. Index date was defined as the first date of P2Y_12_-I initiation. Veterans were followed for sepsis that was severe enough to require hospitalization by patients given P2Y_12_-Is and the overall risk of developing sepsis based on the type of P2Y_12_-I used. Sepsis was ascertained from the primary discharge diagnosis using the International Classification of Diseases, Ninth and Tenth Revisions Clinical Modification and the Diagnosis Related Groups codes. Patients were right-censored if they had no sepsis event by the end of follow-up (September 30, 2019), died without a sepsis event, or survived until the end of the follow-up. Loss to follow-up was also accounted by censoring individuals at their last recorded date of database activity. Propensity score was generated by logistic regression for baseline differences between groups, and we used propensity score overlap weighting to adjust for such differences in Cox proportional hazard models to examine the association of more potent P2Y12-I (ticagrelor or prasugrel) versus the less potent clopidogrel with sepsis (see Supplemental Methods for further details).

The final cohort comprised 55,349 patients with incident CKD who received P2Y_12_-Is: 96.7% of the patients were in the clopidogrel group (*n*=53,524) and the remaining 3.3% of patients were in the prasugrel or ticagrelor group (*n*=1825) (Supplemental Figure 1). The median age for the clopidogrel group was 64 years, and the median age for the prasugrel or ticagrelor group was 62 years. Overall, 98% of patients were men, 13.0% were Black, and 5.5% were other races. A more extensive list of baseline characteristics of the two groups is presented in Supplemental Table 1. There were 11,590 sepsis-related hospitalizations during a median follow-up of 2.6 years. Patients who received prasugrel or ticagrelor were hospitalized with sepsis less often than patients who received clopidogrel (Figure 1A). Overall, we observed a 24% lower risk in hospitalization from sepsis among patients who received prasugrel or ticagrelor instead of clopidogrel. This finding remained statistically significant even after propensity score weighting (Figure 1B).

**Figure 1:**
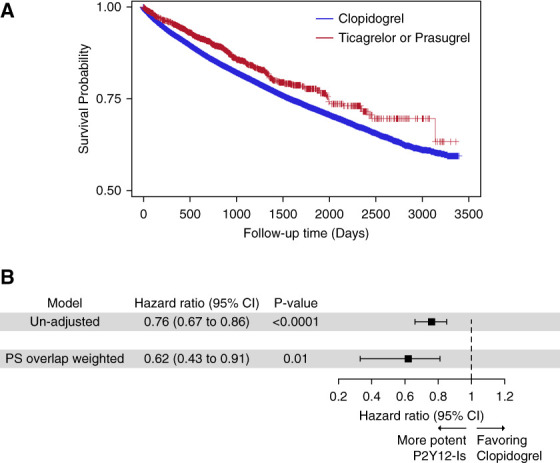
**Risk of being hospitalized with sepsis in patients with incident CKD who were treated with either prasugrel, ticagrelor, or clopidogrel.** (A) Kaplan-Meier curve of the probability that patients with incident CKD survive when hospitalized with sepsis based on treatment with either more potent P2Y_12_-Is (red) or clopidogrel (blue). (B) Forest plot of hazard ratios with 95% CIs for patients on more potent P2Y_12_-Is versus patients on clopidogrel in unadjusted, PS weighted Cox models. Follow-up time was 166.92 per 1000 person-years in the clopidogrel group and 4.82 per 1000 person-years in the prasugrel or ticagrelor group. CI, confidence interval; P2Y_12_-I, P2Y_12_ inhibitor; PS, propensity score.

Thus, we report that targeting the platelet P2Y_12_ receptor with more potent antiplatelet therapies reduced the risk of sepsis-related hospitalization in patients with CKD who were not receiving dialysis. These findings are consistent with our published findings that the use of potent P2Y_12_-Is was associated with lower risk of lethal sepsis in patients on dialysis.^6^ These results further support our recently published hypothesis that platelets, through P2Y_12_ receptors and their inhibitors, modulate inflammation in CKD.^4,7^ In this letter, we also report that sepsis is common in patients with CKD—nearly one in five individuals were hospitalized for sepsis within 2.6 years. This event rate for sepsis is similar to recent reports in Medicare beneficiaries—using methods similar to ours—to ascertain the outcome of sepsis.^2,3^ Despite a few limitations, specifically the use of retrospective data that may suffer from residual confounding, predominantly male Veteran population, assessment of exposure only at baseline, and differences in drug effects based on clinical indication for their use, our results are innovative. This novel hypothesis opens new avenues for researching and treating CKD, especially investigating use of potent P2Y_12_-I in reducing sepsis-related complications in patients with CKD.

## Supplementary Material

## Data Availability

Data cannot be shared. The data were provided for VA researcher C.P. Kovesdy by VA Information Resource Center. Data supporting the findings of this study are available in an intranet site to researchers with VA network access. Intranet version of this page is available at https://vaww.virec.research.va.gov/RUGs/RUGs-Index.htm. VA Information Resource Center is the data custodian for the current research project involving VA/Centers for Medicare & Medicaid Services data.

## Supplemental Material

Supplemental Methods

Supplemental Figure 1. Flow chart of patient selection.

Supplemental Table 1. Baseline characteristics of the cohort.
