# Supplementary material for: Potent P2Y12 Receptor Inhibitors Reduce Risk for Hospitalization from Sepsis in Patients with CKD
Source: Kidney360. 2025 Jul 21;6(11):1994–6. doi: 10.34067/KID.0000000918 (PMC12626676; doi:10.34067/KID.0000000918)
Supplement: Supplementary file 1 [file kidney360-6-1994-s001.pdf]

ASN American Society of Nephrology | JASN Journal of the American Society of Nephrology | KIDNEY360 Accessing Our World From Every Angle | CJASN Clinical Journal of the American Society of Nephrology

ASN Journal Disclosure Form

As per ASN journal policy, I have disclosed any financial relationships or commitments I have held in the past 36 months as included below. I have listed my Current Employer below to indicate there is a relationship requiring disclosure. If no relationship exists, my Current Employer is not listed.

D. Jadvani reports the following:
Employer: University of Arkansas for Medical Sciences

I understand that the information above will be published within the journal article, if accepted, and that failure to comply and/or to accurately and completely report the potential financial conflicts of interest could lead to the following: 1) Prior to publication, article rejection, or 2) Post-publication, sanctions ranging from, but not limited to, issuing a correction, reporting the inaccurate information to the authors' institution, banning authors from submitting work to ASN journals for varying lengths of time, and/or retraction of the published work.

Name: Dharmik Jadvani
Manuscript ID: K360-2025-000208R1
Manuscript Title: Potent P2Y12 Receptor Inhibitors Reduce Risk for Hospitalization from Sepsis in Patients with Chronic Kidney Disease
Date of Completion: June 10, 2025
Disclosure Updated Date: June 10, 2025

ASN American Society of Nephrology | JASN Journal of the American Society of Nephrology | KIDNEY360 Accessing Our World From Every Angle | CJASN Clinical Journal of the American Society of Nephrology

ASN Journal Disclosure Form

As per ASN journal policy, I have disclosed any financial relationships or commitments I have held in the past 36 months as included below. I have listed my Current Employer below to indicate there is a relationship requiring disclosure. If no relationship exists, my Current Employer is not listed.

N. Jain reports the following:
Employer: University of Arkansas for Medical Sciences; and Research Funding: Dialysis Clinic Inc.

I understand that the information above will be published within the journal article, if accepted, and that failure to comply and/or to accurately and completely report the potential financial conflicts of interest could lead to the following: 1) Prior to publication, article rejection, or 2) Post-publication, sanctions ranging from, but not limited to, issuing a correction, reporting the inaccurate information to the authors' institution, banning authors from submitting work to ASN journals for varying lengths of time, and/or retraction of the published work.

Name: Nishank Jain
Manuscript ID: K360-2025-000208R1
Manuscript Title: Potent P2Y12 Receptor Inhibitors Reduce Risk for Hospitalization from Sepsis in Patients with Chronic Kidney Disease
Date of Completion: June 9, 2025
Disclosure Updated Date: June 9, 2025

ASN American Society of Nephrology
JASN Journal of the American Society of Nephrology
KIDNEY360 Accessing Our World From Every Angle
CJASN Clinical Journal of the American Society of Nephrology

ASN Journal Disclosure Form

As per ASN journal policy, I have disclosed any financial relationships or commitments I have held in the past 36 months as included below. I have listed my Current Employer below to indicate there is a relationship requiring disclosure. If no relationship exists, my Current Employer is not listed.

L. Johnson reports the following:
Employer: University of Arkansas for Medical Sciences

I understand that the information above will be published within the journal article, if accepted, and that failure to comply and/or to accurately and completely report the potential financial conflicts of interest could lead to the following: 1) Prior to publication, article rejection, or 2) Post-publication, sanctions ranging from, but not limited to, issuing a correction, reporting the inaccurate information to the authors' institution, banning authors from submitting work to ASN journals for varying lengths of time, and/or retraction of the published work.

Name: Lucas R. Johnson
Manuscript ID: K360-2025-000208R1
Manuscript Title: Potent P2Y12 Receptor Inhibitors Reduce Risk for Hospitalization from Sepsis in Patients with Chronic Kidney Diseas
Date of Completion: June 10, 2025
Disclosure Updated Date: June 10, 2025

ASN
American Society of Nephrology
JASN
Journal of the American Society of Nephrology
KIDNEY360
Accessing Our World From Every Angle
CJASN
Clinical Journal of the American Society of Nephrology

ASN Journal Disclosure Form

As per ASN journal policy, I have disclosed any financial relationships or commitments I have held in the past 36 months as included below. I have listed my Current Employer below to indicate there is a relationship requiring disclosure. If no relationship exists, my Current Employer is not listed.

C. Kovesdy reports the following:
Employer: Memphis VA Medical Center, University of Tennessee Health Science Center; Consultancy: Ardelyx, Astra Zeneca, Bayer, Boehringer Ingelheim, Cara Therapeutics, CSL Behring, GSK, ProKidney, Renibus, Takeda; Ownership Interest: Beamlink; Research Funding: Astra-Zeneca, Bayer, Novartis; Honoraria: Ardelyx, Astra Zeneca, Bayer, Boehringer Ingelheim, Cara Therapeutics, CSL Behring, GSK, ProKidney, Renibus, Takeda; Patents or Royalties: Royalties from UpToDate, Springer; and Advisory or Leadership Role: Associate editor: Diabetes Care; Editorial board: Kidney Medicine, Kidney International Reports; President Elect: International Society of Renal Nutrition and Metabolism.

I understand that the information above will be published within the journal article, if accepted, and that failure to comply and/or to accurately and completely report the potential financial conflicts of interest could lead to the following: 1) Prior to publication, article rejection, or 2) Post-publication, sanctions ranging from, but not limited to, issuing a correction, reporting the inaccurate information to the authors' institution, banning authors from submitting work to ASN journals for varying lengths of time, and/or retraction of the published work.

Name: Csaba P. Kovesdy
Manuscript ID: K360-2025-000208R2
Manuscript Title: Potent P2Y12 Receptor Inhibitors Reduce Risk for Hospitalization from Sepsis in Patients with Chronic Kidney Disease
Date of Completion: June 30, 2025
Disclosure Updated Date: April 7, 2025

ASN
American Society of Nephrology
JASN
Journal of the American Society of Nephrology
KIDNEY360
Accessing Our World From Every Angle
CJASN
Clinical Journal of the American Society of Nephrology

ASN Journal Disclosure Form

As per ASN journal policy, I have disclosed any financial relationships or commitments I have held in the past 36 months as included below. I have listed my Current Employer below to indicate there is a relationship requiring disclosure. If no relationship exists, my Current Employer is not listed.

P. Shrestha reports the following:
Employer: University of Tennessee Health Science Center; Sanegene Bio Inc.

I understand that the information above will be published within the journal article, if accepted, and that failure to comply and/or to accurately and completely report the potential financial conflicts of interest could lead to the following: 1) Prior to publication, article rejection, or 2) Post-publication, sanctions ranging from, but not limited to, issuing a correction, reporting the inaccurate information to the authors' institution, banning authors from submitting work to ASN journals for varying lengths of time, and/or retraction of the published work.

Name: Prabin Shrestha
Manuscript ID: K360-2025-000208R1
Manuscript Title: Potent P2Y12 Receptor Inhibitors Reduce Risk for Hospitalization from Sepsis in Patients with Chronic Kidney Disease
Date of Completion: June 10, 2025
Disclosure Updated Date: June 10, 2025
