# Supplementary material for: Potent P2Y12 Receptor Inhibitors Reduce Risk for Hospitalization from Sepsis in Patients with CKD
Source: Kidney360. 2025 Jul 21;6(11):1994–6. doi: 10.34067/KID.0000000918 (PMC12626676; doi:10.34067/KID.0000000918)
Supplement: Supplementary file 2 [file kidney360-6-1994-s002.pdf]

SUPPLEMENTAL MATERIALS

Supplemental Methods

Cohort definition

We examined data included in the Therapeutic Interventions in Chronic Kidney Disease study, a historic cohort of 3,562,882 US Veterans that had estimated glomerular filtration rates (eGFRs) ≥ 60 mL/min/1.73 m^2 at the time of cohort enrollment, which ran from October 1, 2004 through September 30, 2006; Veterans were followed until September 30, 2019 [S1]. From this cohort, we identified patients who developed incident chronic kidney disease (CKD) (i.e., patients who had an eGFR < 60 mL/min/1.73 m^2: This eGFR had to be recorded at least twice with more than 90 days between eGFR tests, and the eGFR had to be at least 25% lower than the eGFR determined for the person during cohort enrollment. We then identified patients who were new users of $P2Y_{12}$ inhibitors (P2Y12-Is). New users were defined as patients who received P2Y12-Is for ≥ 30 days and who had not received P2Y12-Is during the 365 days prior to the start of the P2Y12-Is. New users had to have a record of enrollment in the Veteran Affairs (VA) pharmacy throughout this timeframe.

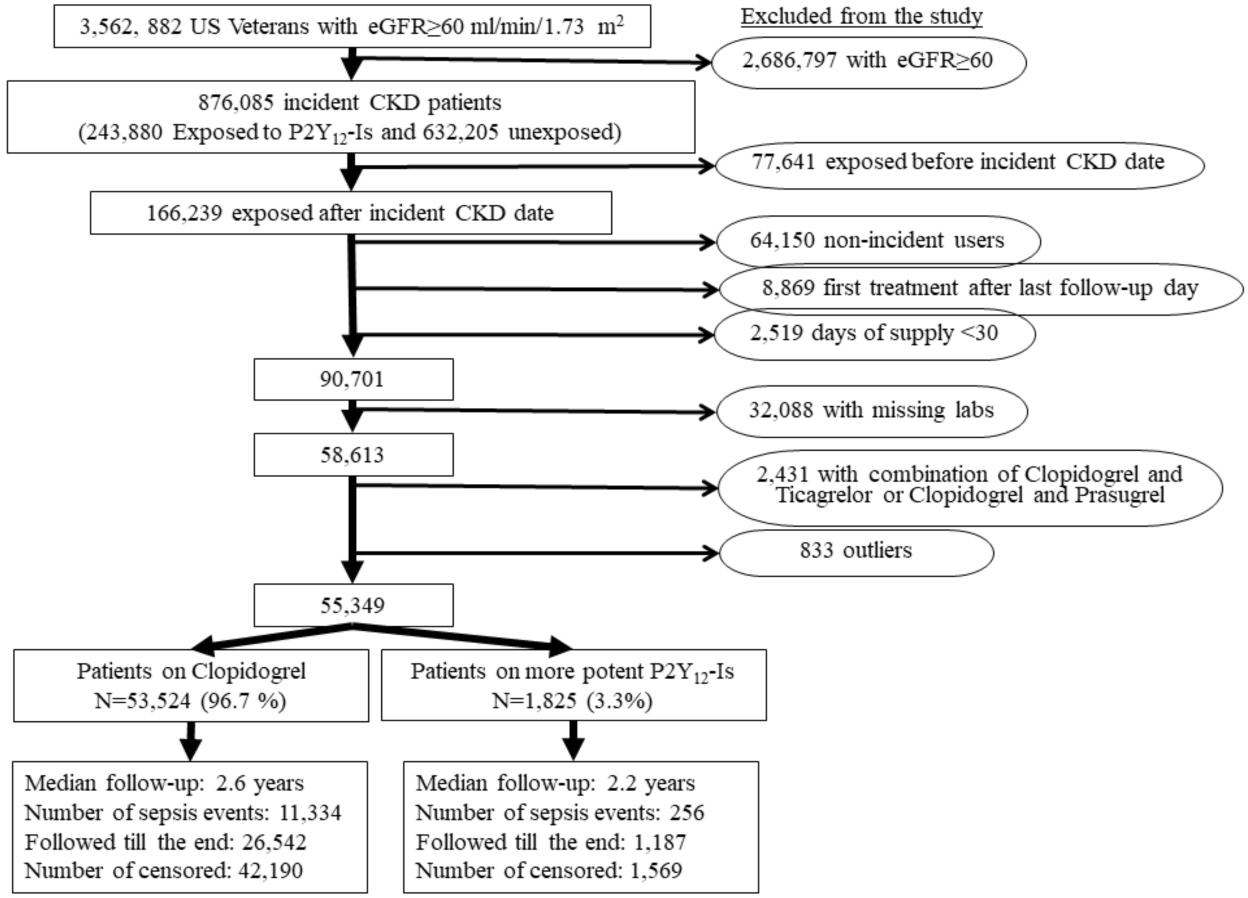

Supplemental Figure 1. Flow chart of patient selection. From the parent cohort of 876,085 patients with incident CKD, 243,880 were exposed to $P2Y_{12}$-Is. Of those, we excluded 77,641 patients who were exposed to the $P2Y_{12}$-Is before being diagnosed with incident CKD.

Among the remaining 166,239 patients, we identified 90,701 patients who used P2Y12-Is for the first time (i.e., new users) who had ≥ 30 days of supply. We then excluded 32,088 patients who were missing information about baseline characteristics and 833 patients with unusual eGFR, platelet, body mass index, and hemoglobin measurements. Finally, we excluded 2,431 (< 0.1%) patients who received a combination of clopidogrel and ticagrelor or clopidogrel and prasugrel. Thus, our final study sample consisted of 55,349 patients with eGFR < 60 mL/min/1.73 m^2: 53,524 (97%) were given clopidogrel and 1,825 (3%) were given potent $P2Y_{12}$-Is (i.e., ticagrelor or prasugrel), **see Table S1**. Of the final cohort, 89% of the members also had either albuminuria or proteinuria data available.

Exposure and outcomes

We used an incident new user design to define treatment exposure. Patients were considered incident new users if they received a dispensation of ≥ 30 days of a $P2Y_{12}$-I and if they did not receive the same medication at any time during the prior 365 days. First date of prescription was the index date. Incident new users also had to have a record of VA pharmacy enrollment. The exposure variable was 2 different types of $P2Y_{12}$-Is: clopidogrel versus more potent $P2Y_{12}$-Is (i.e., prasugrel or ticagrelor). The 2 exposure groups were balanced for their baseline differences with weights generated from propensity scores.

The incident new users were followed from index date until the outcome, the last recorded VA encounter date or the end of follow-up. We analyzed associations of $P2Y_{12}$-Is with the outcome. To do so, we used an intention-to-treat-like design where the treatment exposure at cohort entry is carried forward irrespective of future treatment status. We compared patients who started clopidogrel with patients who started the more potent $P2Y_{12}$-Is ticagrelor or prasugrel.

Sepsis was defined via International Classification of Diseases codes from hospitalization claims based on primary discharge diagnoses of sepsis from any cause. The table below shows codes used for outcome ascertainment, similar to codes used in recent publications by other investigators:[3,8,9]

ICD-9 Code	Description from https://www.cdc.gov/nchs/data/icd/icd9cm_guidelines_2011.pdf
995.92	Severe sepsis
995.91	SIRS due to infectious process without organ dysfunction
038.xx	Systemic infection
998.02	Post-operative septic shock
785.52	Septic shock
**ICD-10 codes**	
A400	Sepsis due to streptococcus, group A
A401	Sepsis due to streptococcus, group B
A403	Sepsis due to Streptococcus pneumoniae
A408	Other streptococcal sepsis
A409	Streptococcal sepsis, unspecified
A4101	Sepsis due to methicillin susceptible Staphylococcus aureus
A4102	Sepsis due to methicillin resistant Staphylococcus aureus
A411	Sepsis due to other specified staphylococcus
A412	Sepsis due to unspecified staphylococcus
A413	Sepsis due to Hemophilus influenzae

A414	Sepsis due to anaerobes
A4150	Gram-negative sepsis, unspecified
A4151	Sepsis due to Escherichia coli [E. coli]
A4152	Sepsis due to Pseudomonas
A4153	Sepsis due to Serratia
A4159	Other Gram-negative sepsis
A4181	Sepsis due to Enterococcus
A4189	Other specified sepsis
A419	Sepsis, unspecified organism
**DRG codes for sepsis**	
548	SEPTIC ARTHRITIS W MCC
549	SEPTIC ARTHRITIS W CC
550	SEPTIC ARTHRITIS W/O CC/MCC
870	SEPTICEMIA OR SEVERE SEPSIS W MV >96 HOURS
871	SEPTICEMIA OR SEVERE SEPSIS W/O MV >96 HOURS W MCC
872	SEPTICEMIA OR SEVERE SEPSIS W/O MV >96 HOURS W/O MCC
288	ACUTE & SUBACUTE ENDOCARDITIS W MCC
289	ACUTE & SUBACUTE ENDOCARDITIS W CC
290	ACUTE & SUBACUTE ENDOCARDITIS W/O CC/MCC

Data collection

We obtained information about baseline demographic characteristics, comorbidities, medications, vital signs, and laboratory variables from the Veterans Affairs (VA) Corporate Data Warehouse (CDW) [S2]. Medication data were extracted from outpatient and inpatient pharmacy files from the Decision Support System National Data Extracts and from Medicare Part D files [S3]. Information about medications received from non-VA sources was obtained from non-VA medication files in CDW; this included over the counter medications, herbal supplements, VA-prescribed medications filled at non-VA pharmacies, and medications prescribed by providers outside the VA. We defined concomitant medication use as the presence of at least 1 outpatient dispensation of ≥ 30 days during the 365 days prior to the index date. Comorbidities were identified from the VA Inpatient and Outpatient Medical SAS Datasets based on the presence of International Classification of Disease (ICD)-9 and ICD-10 diagnostic and procedure codes and Current Procedural Terminology codes, as well as from Centers for Medicare and Medicaid Services Data files. We used the Deyo modification for administrative data sets to calculate the Charlson Comorbidity Index [S4]. Laboratory data were obtained from the VA LabChem files [S5], and eGFR was estimated from the 2009 Chronic Kidney Disease Epidemiology Collaboration equation [S6]. Urine albumin-to-creatinine ratios were taken as the most recent ones and 1 prior to baseline date (similar to eGFR values). Urine protein to creatinine ratio and urine dipstick protein values were converted to urine albumin-to-creatinine ratio values using the conversion equations [S8].

Statistical analysis

We described data as number (%) for categorical variables and mean ± standard deviation or median (25th–75th percentile), as appropriate. We used standardized differences to compare characteristics between patients given clopidogrel and patients given ticagrelor or prasugrel. Using multivariate Cox proportional hazard models, we examined the association of ticagrelor or

prasugrel against clopidogrel when patients had sepsis. We used logistic regression models to calculate propensity scores (PS) from baseline characteristics listed below.

Demographics: age (years), gender (male), race (White, African American, and Others), smoker (current, past, never, or other), service-connected (yes or no), insurance type (none, Medicare, other).

Concomitant medicines on index date: antihypertensives (i.e., RAAS inhibitors, thiazide diuretics, loop diuretics, potassium sparing diuretics, other antihypertensives), NSAIDs, SGLT2i inhibitors, proton pump inhibitors, mineralocorticoids, corticosteroids, statins, antihistamine, aspirin, antiplatelet (other than aspirin), hydrogen receptor blockers, calcineurin inhibitors.

Comorbidities: diabetes mellitus, dementia, hepatocellular carcinoma, hepatitis C, hypertension, liver cirrhosis, liver transplant, liver disease, chronic pulmonary disease, congestive heart failure, cerebrovascular disease, peripheral vascular disease, cancer, metastatic cancer, HIV/AIDS, ischemic heart disease, myocardial infarction, stomach ulcer, bipolar disorder, major depression, psychotic disorders, and post-traumatic stress disorder.

Laboratory data on index date: eGFR, body mass index, platelets, proteinuria and hemoglobin.

We used a PS-overlap weighting method [S8, S9] to account for differences in baseline characteristics between the 2 patient treatment groups. This weighting scheme makes observations with a substantial probability for either treatment (based on the PS model) more influential and smoothly down-weights patients in the tails of the PS distribution, thus mitigating undue influence of patients in either treatment that were unlikely candidates for the respective other treatment (without the need to exclude them altogether based on arbitrary cut-off values). Analyses were conducted using Stata MP version 17.1 (StataCorp, College Station, TX, USA) and SAS 9.4 (SAS Institute Inc., Cary, NC, USA). The study was approved by the Institutional Review Boards of the Memphis and Long Beach VA Medical Centers, with exemption from informed consent.

Supplemental References

S1. Hassan W, Shrestha P, Sumida K et al. Association of uric acid-lowering therapy with incident chronic kidney disease. JAMA Netw Open 2022;5:e2215878. https://doi.org/10.1001/jamanetworkopen.2022.15878

S2. VA Information Resource Center. VIReC Resource Guide: VA Corporate Data Warehouse. Hines, IL: US Department of Veterans Affairs, Health Services Research and Development Service, VA Information Resource Center, 2012.

S3. US Department of Veterans Affairs, VA Information Resource Center (VIReC). VIReC Research User Guide: VHA Pharmacy Prescription Data, 2nd edn. Hines, IL: VIReC, 2008.

S4. Deyo RA, Cherkin DC, Ciol MA. Adapting a clinical comorbidity index for use with ICD-9-CM administrative databases. J Clin Epidemiol 1992;45:613–619. https://doi.org/10.1016/0895-4356(92)90133-8

S5. Kovesdy CP, Alrifai A, Gosmanova EO et al. Age and outcomes associated with BP in patients with incident CKD. Clin J Am Soc Nephrol 2016;11:821–831. https://doi.org/10.2215/CJN.08660815

S6. Levey AS, Stevens LA, Schmid CH et al. A new equation to estimate glomerular filtration rate. Ann Intern Med 2009;150:604–612. https://doi.org/10.7326/0003-4819-150-9-200905050-00006

S7. Sumida K, Nadkarni GN, Grams ME, et al. Conversion of Urine Protein-Creatinine Ratio or Urine Dipstick Protein to Urine Albumin-Creatinine Ratio for Use in Chronic Kidney Disease Screening and Prognosis: An Individual Participant-Based Meta-analysis. Ann Intern Med 2020;173(6):426-435.

S8. Li F, Thomas LE, Li F. Addressing extreme propensity scores via the overlap weights. Am J Epidemiol 2019;188:250–257.

S9. Thomas LE, Li F, Pencina MJ. Overlap weighting: a propensity score method that mimics attributes of a randomized clinical trial. JAMA 2020;323:2417–2418.

Supplemental Table 1. Baseline characteristics of the cohort

	All<br>*(n = 55,349)*	Clopidogrel Users<br>*(n = 53,524)*	Prasugrel or Ticagrelor Users<br>*(n = 1,825)*	Standardized Difference
Age (in years)	73.0 ± 9.1	73.1 ± 9.1	70.9 ± 8.0	0.00
Gender (male), n (%)	54,117 (98)	52,342 (98)	1,775 (97)	0.00
Race, n (%)				0.00
White	44,879 (81)	43,389 (81)	1,490 (82)	
African American	7,448 (13)	7,205 (13)	243 (13)	
Other	3,022 (5.5)	2,930 (5.5)	92 (5.0)	
Smoker, n (%)				0.00
Current	25,124 (45)	24,313 (45)	811 (44)	
Past	11,493 (21)	11,137 (21)	356 (20)	
Never	9,805 (18)	9,474 (18)	331 (18)	
Other	8,927 (16)	8,600 (16)	327 (18)	
Service-connected, n (%)	29,175 (53)	28,141 (53)	1,034 (57)	0.00
Insurance type, n (%)				0.00
None	7,864 (14)	7,594 (14)	270 (15)	

Medicare	43,314 (78)	41,927 (78)	1,387 (76)	
Other	4,171 (7.5)	4,003 (7.5)	168 (9.2)	
RAAS inhibitors, n (%)	45,054 (81)	43,511 (81)	1,543 (85)	0.00
Thiazide diuretics, n (%)	22,606 (41)	21,904 (41)	702 (38)	0.00
Loop diuretics, n (%)	28,710 (52)	27,807 (52)	903 (49)	0.00
Potassium sparing diuretics, n (%)	9,736 (18)	9,384 (18)	352 (19)	0.00
Other antihypertensives, n (%)	49,637 (90)	47,915 (90)	1,722 (94)	0.00
NSAIDs, n (%)	40,440 (73)	39,136 (73)	1,304 (71)	0.00
SGLT2i inhibitors, n (%)	1,466 (2.6)	1,376 (2.6)	90 (4.9)	0.00
Proton pump inhibitors, n (%)	35,142 (63)	33,975 (63)	1,167 (64)	0.00
Mineralocorticoids, n (%)	9,222 (17)	8,885 (17)	337 (18)	0.00
Corticosteroids, n (%)	2,226 (4.0)	2,146 (4.0)	80 (4.4)	0.00
Statins, n (%)	51,987 (94)	50,237 (94)	1,750 (96)	0.00
Antihistamine, n (%)	46,457 (84)	44,970 (84)	1,487 (81)	0.00
Aspirin, n (%)	33,248 (60)	32,203 (60)	1,045 (57)	0.00
Antiplatelet (other than aspirin), n (%)	55,004 (99)	53,195 (99)	1,809 (99)	0.00
Hydrogen receptor blockers, n (%)	13,764 (25)	13,369 (25)	395 (22)	0.00
Calcineurin inhibitors, n (%)	276 (0.5)	267 (0.5)	9 (0.5)	0.00

Diabetes mellitus, n (%)	21,825 (39)	21,145 (40)	680 (37)	0.00
Dementia, n (%)	339 (0.6)	331 (0.6)	8 (0.4)	0.00
Hepatocellular carcinoma, n (%)	5 (0.01)	5 (0.01)	0 (0)	0.00
Hepatitis C, n (%)	1,583 (2.9)	1,545 (2.9)	38 (2.1)	0.00
Hypertension, n (%)	37,828 (68)	36,679 (69)	1,149 (63)	0.00
Liver cirrhosis, n (%)	217 (0.4)	211 (0.4)	6 (0.3)	0.00
Liver transplant, n (%)	40 (0.07)	39 (0.07)	1 (0.05)	0.00
Liver disease, n (%)	1,393 (2.5)	1,357 (2.5)	36 (2.0)	0.00
Chronic pulmonary disease, n (%)	7,821 (14)	7,616 (14)	205 (11)	0.00
Congestive heart failure, n (%)	3,493 (6.3)	3,399 (6.4)	94 (5.2)	0.00
Cerebrovascular disease, n (%)	4,908 (8.9)	4,833 (9.0)	75 (4.1)	0.00
Peripheral vascular disease, n (%)	4,619 (8.3)	4,520 (8.4)	99 (5.4)	0.00
Cancer, n (%)	4,528 (8.2)	4,425 (8.3)	103 (5.6)	0.00
Metastatic cancer, n (%)	158 (0.3)	156 (0.3)	2 (0.1)	0.00
HIV/AIDS, n (%)	281 (0.5)	273 (0.5)	8 (0.4)	0.00
Ischemic heart disease, n (%)	17,608 (32)	17,061 (32)	547 (30)	0.00
Myocardial infarction, n (%)	4,074 (7.4)	3,952 (7.4)	122 (6.7)	0.00
Ulcer, n (%)	1,105 (2.0)	1,067 (2.0)	38 (2.1)	0.00

Bipolar disorder, n (%)	1,376 (2.5)	1,326 (2.5)	50 (2.7)	0.00
Major depression, n (%)	3,453 (6.2)	3,324 (6.2)	129 (7.1)	0.00
Psychotic disorders, n (%)	1,400 (2.5)	1,360 (2.5)	40 (2.2)	0.00
PTSD, n (%)	4,927 (8.9)	4,745 (8.9)	182 (10.0)	0.00
Charlson comorbidity index, median (25th–75th percentile)	1 (0–2)	1 (0–2)	1 (0–2)	0.00
eGFR, ml/min/1.73m^2, n (SD)	57.2 ± 16.6	57.2 ± 16.6	56.6 ± 15.9	0.00
Body mass index, kg/m^2, n (SD)	29.7 ± 5.8	29.7 ± 5.8	30.6 ± 5.9	0.00
Platelet count, k/mm^3, n (SD)	5.3 ± 0.3	5.3 ± 0.3	5.3 ± 0.3	0.00
Hemoglobin, g/dl, n (SD)	13.2 ± 1.8	13.1 ± 1.8	13.3 ± 1.8	0.00

Abbreviations: eGFR, estimated glomerular filtration rate; HIV/AIDS, Human Immunodeficiency Virus/Acquired Immunodeficiency Syndrome; NSAIDs, Non-Steroidal Anti-Inflammatory Drugs; PTSD, post-traumatic stress disorder; RAAS, Renin Angiotensin Aldosterone System; SGLT2i, Sodium-Glucose Cotransporter-2 inhibitor.
